# The effect of heat pre-treatment on the anaerobic digestion of high-solid pig manure under high organic loading level

**DOI:** 10.3389/fbioe.2022.972361

**Published:** 2022-11-03

**Authors:** Pengfei Li, Jianlin Wang, Hao Peng, Qichen Li, Ming Wang, Wencong Yan, Stopira Yannick Benz Boboua, Wenzhe Li, Yong Sun, Guoxiang Zheng, Hongqiong Zhang

**Affiliations:** ^1^ College of Engineering, Northeast Agricultural University, Harbin, China; ^2^ Key Laboratory of Combining Farming and Animal Husbandry, Heilongjiang Academy of Black Soil Conservation and Utilization, Ministry of Agriculture, Harbin, China; ^3^ Institute of Environment and Sustainable Development in Agriculture, Chinese Academy of Agricultural Sciences (CAAS), Beijing, China; ^4^ Key Laboratory of Pig-breeding Facilities Engineering, Ministry of Agriculture and Rural Affairs, Harbin, China; ^5^ Huanghe Science and Technology College, Zhengzhou, China

**Keywords:** heat pretreatment, biogas production, energy balance, organic waste, pig manure

## Abstract

Since more and more large-scale farms appear in China and changes in fecal sewage source disposal, the production of high-concentration solid manure waste is also increasing, and its conversion and utilization are gaining attention. This study investigated the effect of heat pre-treatment (HPT) on the thermophilic anaerobic digestion (AD) of high-solid manure (HSM). Pig manure (PM) feed with a total solids of 13% was used for the HPT and subsequent anaerobic digestion (AD) test. The HPT was carried out at 60°C, 80°C, and 100°C, respectively, for 15 min after the heating reached the set temperature. The results show that HPT led to PM feed COD solubilization, observing a maximum increase of 24.57% after pretreated at 100°C, and the treated PM feed under this condition received the maximum methane production potential of 264.64 mL·g^−1^ VS in batch AD test, which was 28.76% higher than that of the untreated group. Another semi-continuous AD test explored the maximum volume biogas production rate (VBPR). It involves two organic loading rates (OLR) of 13.4 and 17.8 g VS_added_·L^−1^·d^−1^. The continuous test exhibited that all the HPT groups could produce biogas normally when the OLR increased to the high level, while the digester fed with untreated PM showed failure. The maximum VBPR of 4.71 L L^−1^·d^−1^ was observed from PM feed after pre-treated at 100°C and running at the high OLR. This reveals that thermal treatment can weaken the impact of a larger volume of feed on the AD system. Energy balance analysis demonstrates that it is necessary to use a heat exchanger to reuse energy in the HPT process to reduce the amount of energy input. In this case, the energy input to energy output (*E*_*i*_/*E*_*o*_) ranged from 0.34 to 0.55, which was much less than one, suggesting that biogas increment due to heat treatment can reasonably cover the energy consumption of the pre-treatment itself. Thus combining HPT and high-load anaerobic digestion of PM was suitable.

## Introduction

In recent years, more large-scale modern pig farms have emerged in China to improve management efficiency and ensure food safety. However, the pressure of centralized manure treatment is also increasing (Qian et al., 2018; Pan et al., 2021). Both the methods of dry-wet separation (DYS) collection and mechanical extrusion dehydration are widely used in the initial treatment of manure. As a result, these farms generate two types of waste: low-solids wastewater (LSW) and high-solids manure (HSM). LSW can be discharged or reused after biochemical, filtration, and disinfection treatments. The solid content of HSM is generally above 15%. It contains a large amount of animal digestive waste and is the primary source of pollution. Thus the treatment or conversion of HSM has been the focus of attention in large-scale farms. Since HSM’s water content is still higher than 80%, it is not ideal for natural composting conditions. However, it can be an excellent feedstock for anaerobic digestion (AD) (Wang et al., 2021).

AD is widely recognized and utilized as an important form of organic matter conversion because of its ability to produce biogas (CH_4_ + CO_2_). Biogas can be used as a sustainable energy source to reduce carbon emissions from fossil energy (Awe et al., 2017). Many studies have revealed that high-solid anaerobic digestion (HSAD) has higher biogas production than digestion under low-solid AD mode. Jha reported that the biogas production efficiency and volatile solids (VS) removal rate obtained by the dry fermentation process (total solids (TS) = 15.18%) were higher than those obtained by wet fermentation (TS = 7.68%) (Jha et al., 2013). Wu also found that the methane yield could be improved by up to 39.5% when the AD process was run in a high-solid mode (Wu et al., 2017). Our previous study has also shown that feeding with food waste at a solid ratio of 19.0% resulted in a significantly higher biogas production efficiency than feeding food waste with a solids content of 9.5% (Wang et al., 2015). In addition, at the same organic loading rate (OLR), HSAD tends to have smaller volumes of daily influent and effluent, indicating less consumption of water, heat, and microbes from the tank, which is more favorable for the production of biogas or energy. That is to say, HSAD can easily create a high OLR under small feed and discharge volume with a small impact on the reactor. At the same time, an appropriate increase in organic loading rate (OLR) could improve the volumetric biogas production rate (VBPR), which has been validated in the AD of food waste with a high OLR ranging from 7 to 14 g VS.·L^−1^·d^−1^ (Zhang et al., 2015; Liu et al., 2017; Tassakka et al., 2019). And a higher VBPR can better balance the insulation cost per cubic meter of gas output and thus reduce operating costs (Zhou et al., 2022). In the past, Chinese farms were dominated by large amounts of water flushing operations, which produced manure waste with low solids and promoted the leaching of nutrients, resulting in low yields of HSM. This may be an important reason why HSAD had not received much attention in the AD of manure waste.

Many previous studies have reported that some pre-treatment methods can improve biogas production in the AD process. Such as heat (Ferrer et al., 2008; Kim et al., 2015), ultrasound (Castrillón et al., 2011), advanced oxidation (Almomani et al., 2019), alkaline cracking, dry milling, steam explosion (Fjørtoft et al., 2019), etc. Most of these methods have been proven to be effective. Still, they are challenging to be used in practical engineering due to equipment investment, chemical reagent consumption, and high energy consumption (Orlando and Borja, 2020), which has limited the promotion of pre-treatment technology. Thus, reducing the energy input has been a hot direction in pre-treatment research.

Heat pre-treatment (HPT) at atmospheric pressure at a temperature below 100°C is a gentle and low energy consumption method and is more suitable for the pre-treatment of materials not rich in cellulosic matters, such as sewage sludge (Liao et al., 2016; Liu et al., 2021), food waste (Ariunbaatar et al., 2015), kitchen waste (Li and Jin, 2015) and manure (Lin et al., 2021). Sutaryo revealed that the methane production of pig manure (PM) and dehydrated PM could be increased respectively by 9.5%–22.5% and 6.1%–25.3% when the raw materials were treated by HPT at the temperature from 65°C to 80°C before input (Sutaryo et al., 2014). Passos reported that pre-treatment of microalgae at 95°C for 10 hours could increase the VS solubilization by 1,188%, the initial methane production rate by 90%, and the final methane yield by 60% compared with the control (Passos et al., 2013). Liu demonstrated that the maximum methane production of low-organic content sludge could reach around 294.73 ml g^−1^ VS after 36 h of 90°C pre-treatment, which is 5.56 folds that of the untreated sludge (Liu et al., 2021). Additionally, less equipment investment and lower operation requirements confer HPT with great popularization and application potential.

Although HPT has been proven to improve the specific methane yield, few studies have focused on the HPT application to enhance biogas production both in HSAD and high OLR mode. Besides, the relationship between HPT’s energy input and AD’s energy output has received less attention in previous studies, which is directly related to practical applications’ economics. Therefore, this work aims to evaluate the effect of HPT below 100°C on PM AD performance and energy balance. Methanation potential and kinetics are investigated in a batch test. In contrast, another continuous anaerobic AD test mainly observes the possibility of obtaining the maximum VBPR and discusses the energy balance problem.

## Materials and methods

### Materials

The PM used in this study was collected from a pig farm in the Harbin suburbs, China, where the DYS cleaning mode was adopted for manure collection. The produced dry PM from the farm had a TS above 15%. The inoculum was taken from a continuous stirred tank reactor (CSTR) which had only been used for PM digestion in our laboratory. The reactor operated in semi-continuous AD mode. Its total volume, working volume, operation temperature, pH, hydraulic retention time (HRT), and total solids (TS) content were 15 L, 10 L, 55 ± 1°C, 7.48 ± 0.12, 20 days, 41.5 ± 1.8 g L^−1^, respectively. Before the inoculum was used, it should be fermented for a few days to confirm that it does not produce biogas. The collected PM and inoculum were stored in a 4°C refrigerator before use. The characteristics of the prepared PM and the inoculum are shown in Table 1.

**TABLE 1:** Characteristics of PM and inoculum.

Parameters	pH	TS (%)	VS (%)^a^	TOC (%)^a^	TKN (%)^a^	C/N ratio	SCOD (mg·L^−1^)
PM	7.45	24.1	82.3	42.5	2.91	14.6	20320
Inoculum	7.85	6.42	76.4	36.3	1.89	19.3	3,425
PM feed^b^	7.34	13.0	—	—	—	—	15090

^a^ On a dry basis.

^b^ Obtained by dilution of raw pig manure and used for HTPT and AD test.

### HPT process

Before heat pre-treatment, the raw PM was mixed with tap water into a TS of 13%, which concentration was at a medium-to-high level and could be pumped and mixed on a large scale, more conducive to practical engineering. The temperature of HPT was set at three levels, including 60°C, 80°C, and 100°C. The heating process was carried out with a glass beaker (2 L) placed in an oil bath, stirring during heating, and maintained for 15 min when the temperature reached the desired value. Then, the hot PM feed was cooled down to the fermentation temperature before being put into the digester.

### Biochemical methane potential test

The BMP test was carried out with an automatic methane test system (AMPTS-II, Sweden), and the volume of each reactor was 500 ml. This test involved four groups, including three pre-treatment groups and one untreated group (set as the control). All groups were tested simultaneously, and each was performed in three replicates for 12 bottles. Before start-up, 300 ml of inoculum and 100 ml of PM feed were added to each reactor, and then these reactors were purged with nitrogen gas for 1 minute to remove oxygen fully. The used PM feed TS, the substrate inoculum ratio, the working volume, and the digestion temperature were 13%, 1:3 (V/V), 400 ml, and 55 ± 1°C, respectively. The pH was not controlled, and the running time was more than 20 days. The data fitting of biogas production was conducted with the modified Gompertz model, as shown below (Syaichurrozi et al., 2020).

$$y=A\exp\left\{-\exp\left(\frac{\mu_{m}e}{A}\left(\lambda -t\right)+1\right)\right\}$$ (1)

In the model (1), *y* is the cumulative biogas production on day t (mL∙g^−1^); *t* is the fermentation time (d); *A* is the maximum methane yield (mL∙g^−1^); *μ*_m_ is the maximum daily gas production rate (mL∙g^−1^∙d^−1^); *λ* is the delay time of gas production (d); and e is the natural constant (2.718,282).

### Semi-continuous AD test

The semi-continuous AD process was executed by a CSTR digester with a total volume of 9 L and a working volume of 6 L. The CSTR was kept warm with a hydrothermal jacket, and its feed pipe extended from the side to the internal and below the liquid level to ensure the sealing of the reactor, while the discharge port was located at the bottom of the reactor and controlled by a ball valve. The biogas outlet was located at the top of the reactor and connected to a wet gas flowmeter (LMF-1, Qingdao). After coming out of the flowmeter, the gas was collected in a collecting bag (aluminum foil, 5 L). Before running, the reactor was first added with 6 L of inoculum, and then the test was conducted at a low OLR until stable biogas production to activate or rejuvenate the microorganisms in the inoculum to reduce the impact of the inoculum on the subsequent high load test (Wang et al., 2017). The semi-continuous AD test was also operated under thermophilic conditions (55 ± 1°C), and the PM feeds with different treatments were successively fed into the reactor in the order of untreated samples and pre-treated samples at 60°C, 80°C, and 100°C. Since the solid content of the feed was constant, the condition of ultra-high OLR could be constructed only by reducing the HRT (Ariunbaatar et al., 2021), and two HRT levels of 8 days and 6 days were used for creating two ultra-high OLR conditions of 13.4 g VS_added_∙L^−1^∙d^−1^ and 17.8 g VS_added_∙L^−1^∙d^−1^, respectively. After adding each sample, the digester was run twice the time of HRT. The biogas volume and methane content were recorded daily, and the effluent’s physical and chemical parameters were measured before the feed replacement. All the experimental values were obtained from the average values of three parallel tests.

### Energy balance analysis

**FIGURE 1:**
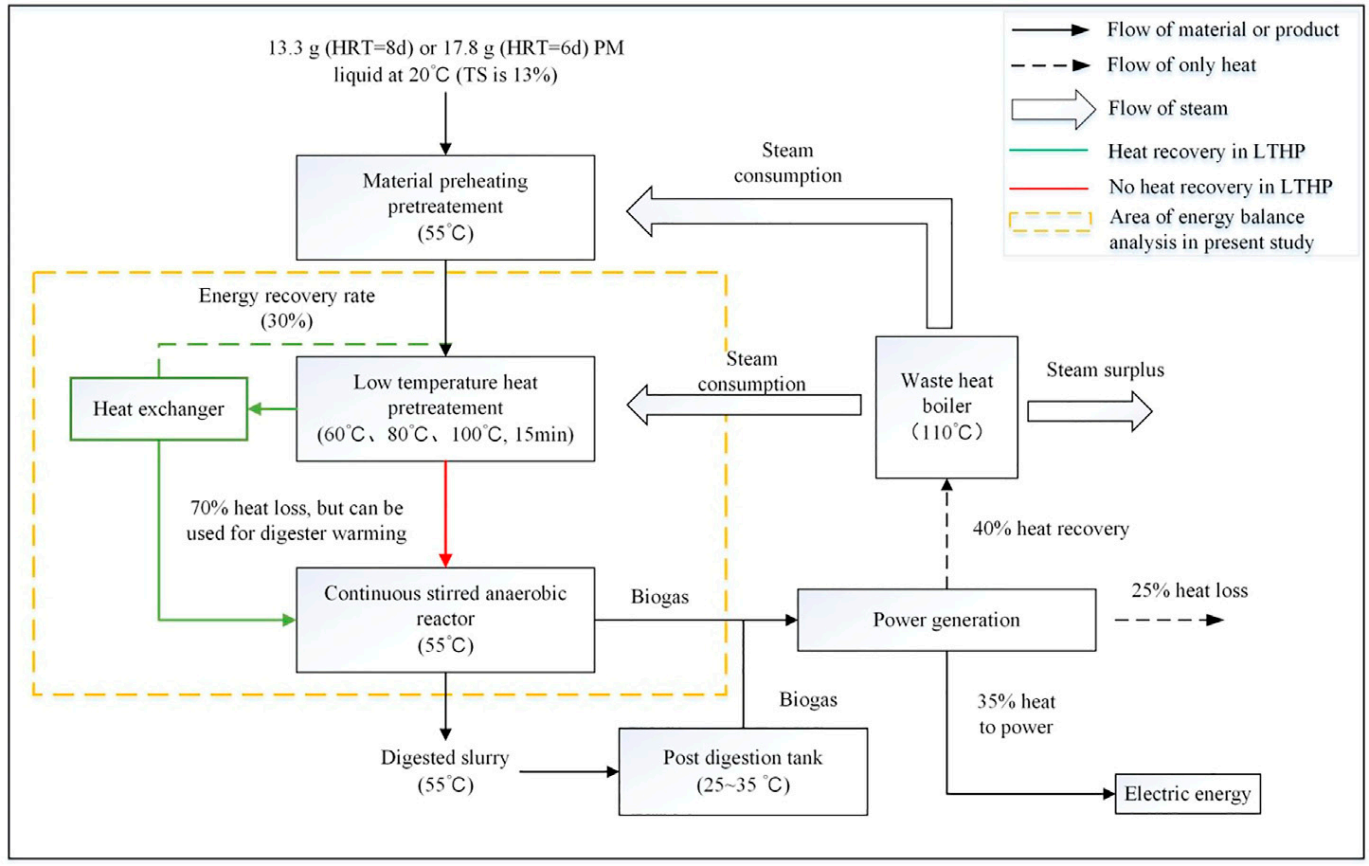
Flows of materials, products and energy of HSAD containing HPT.

Since other operation parameters were consistent, the increased energy input should be mainly consumed in the HPT process. The working volume of the simulated digester was set as 1 L; daily feed and discharge volume was calculated as the working volume divided by HRT, and the operating temperature was set as 55°C. In addition, the whole heating process of fresh materials could be divided into two steps: first, the PM feed was heated from ambient temperature to 55°C and then heated to the pre-treatment temperature. The former could be regarded as necessary energy consumption; thus, it was not included in the benefits analysis for simplification. In addition, due to the short pre-treatment time, the thermal insulation energy consumption could be ignored (Zhou et al., 2021). Figure 1 illustrates the operation of a biogas plant with a heat pre-treatment process. The part in the yellow dashed line clarifies the relationship between energy and material flow in the present simulation assay, and both the heat recovery path (green line) and no heat recovery path (red line) will be discussed.

The ratio of energy input to energy output (*E*_*i*_/*E*_*o*_) under each pre-treatment was considered an indicator of the energy balance. Values lower and equal to 1 represent positive and neutral balance, respectively (Passos et al., 2013; Ometto et al., 2014). The heating energy input (*E*_*i*_) was only related to the energy required for heating the PM feed from 20°C (*T*_*0*_) to the pre-treatment temperature (*T*_*p*_: 60°C, 80°C, and 100°C), which was estimated using Eq. 2. In practice, a general liquid heating process could be carried out by using a heat exchanger for energy saving, and the heat recovery efficiency (*φ*) was assumed to be equal to 50% for 80°C pre-treatment, 60% for 100°C pre-treatment and no heat recovery for 60°C pre-treatment. Because the heated feed liquid only needs to be cooled to 55°C, the heat exchange temperature difference is relatively small, which leads to the fact that the current set *φ* value is lower than the previously reported value of 85% (Lu et al., 2008). While the heat for heating the apparatus was assumed to be negligible, thus the energy output (*E*_*o*_) was calculated from the biogas increment (*ΔP*) multiplied by its heating value (*ξ*). Thus, *E*_*i*_ and *E*_*o*_ can be calculated by Eq. 2 and Eq. 3, respectively.

$$E_{i}=V\times \rho \times \gamma \times \left(T_{p}-T_{0}\right)\times \left(1-\varphi \right)$$ (2)

$$E_{O}=\Delta P\times \xi /1000$$ (3)

In model (2), *E*_*i*_ is the heating energy input (kJ); *V* is the daily PM feed volume (L); *ρ* is the specific density of PM feed and is assumed equal to 1 kg L^−1^; *γ* specific heat value of PM feed and is assumed equal to 4.18 kJ∙(kg∙°C)^−1^; *T*_p_ and *T*_0_ are respectively the temperatures before and after preheating (°C); *φ* is the heat reuse efficiency of the heating process (%). In model (3), *E*_*o*_ is the extra energy output (kJ); *ΔP* is the biogas increment after pre-treatment (L); *ξ* is the lower heating value of biogas (≈18 kJ L^−1^).

### Soluble chemical oxygen demand

SCOD was used to determine the amount of organic matter in the liquid phase. Its growth rate could be used to evaluate the effect of organic matter released from solid particles on the liquid phase under heat pre-treatment. The calculation of solubilization COD was carried out by Eq. 4 (Vlyssides and Karlis, 2004; Şenol et al., 2020);

$$Solubilization\;COD\;\left(\%\right)=\left({SCOD}_{T}-{SCOD}_{0}\right)/\left({COD}_{0}-{COD}_{0}\right)\times 100$$ (4),

where *SCOD*_*0*_ and *COD*_*0*_ are the initial SCOD and COD of PM feed, respectively, and *SCOD*_*T*_ is the SCOD after pre-treatment at each temperature in the unit of mg∙L^−1^.

### Biogas recovery rate (BRR) of semicontinuous AD

In practical biogas production, the most direct way to improve the VBPR is to increase the OLR, which is often achieved by reducing the HRT. Thus, some organic matter with slow degradation may be discharged from the reactor before being completely transformed into biogas. Besides, due to the characteristic of complete mixing of the CSTR, the daily input substrate will be inevitably discharged from the digester to various degrees with the effluent of the next day, which is a continuously ongoing process. As a result, the biogas yield from the CSTR running with semi-continuous AD mode is usually lower than that under the batch mode (such as the BMP test). Therefore, a parameter of BRR was defined in the present study to evaluate the biogas yield from the daily added material in the semi-continuous AD process, which could be calculated by Eq. 5:

$$BRR\left(\%\right)=\left(continuous/y_{\mathrm{BMP}}\right)\times 100$$ (5)

where _continuous_ is the biogas yield obtained from the semi-continuous AD process, mL∙g^−1^ VS_added_; *y*_BMP_ is the maximum methane yield obtained by fitting the data of the BMP test with the modified Gompertz model, mL∙g^−1^ VS.

### Experimental parameters and analytical methods

The biogas composition was determined with a gas chromatograph (GC-6890N, Agilent Inc. United States) equipped with a stainless steel column (1.5 m × 3 mm i.d. Carbon molecular sieve TDX-01: 1.5–2.0 nm) and a thermal conductivity detector (TCD) using argon as the carrier gas. Volatile organic acids (VFAs) were determined by the same GC-6890N equipped with a flame ionization detector (FID) and a capillary column (30 m × 0.25 mm, Agilent 19091N-133) using nitrogen as the carrier gas. The total solids (TS), volatile solids (VS.), pH (Sartorius basic pH meter PB-10, Germany), ammonia nitrogen (AN), total organic carbon (TOC), chemical oxygen demand (COD), and total Kjeldahl nitrogen (TKN) were determined according to standard methods (APHA and AWWA, 2005). Soluble COD (SCOD) of the PM feed was analyzed after vacuum filtration through 0.45 µm membrane filter paper (Borzooei et al., 2021). All measurements were conducted in triplicate, and the averaged data were presented.

## Results and discussion

### Effect of HPT on solubilization

Previous studies have demonstrated that the solubility of some particulate matter in the raw material can be improved by heat pre-treatment. This improvement is mainly ascribed to the promoted dissolution or hydrolysis of the material in a hydrothermal environment, which has been proven to be related to the improvement of biogas production (Kim et al., 2015; Usman Khan and Kiaer Ahring, 2021). Menardo pre-treated dehydrated PM, digested it at 120°C and found that methane production increased by 35%–171%. Increasing soluble COD may be the main reason for improving biogas production of PM after LTPT (Menardo et al., 2011). Huang pre-treated swine manure at 110–130°C for 30 min and achieved a CH_4_ yield of 280.18–328.93 ml g^−1^ VS_fed_ increasing 14%–34%. The reason may be the increase of 13%–26% in soluble organic carbon concentration after pre-treatment (Huang et al., 2017). Bonmatífound that the concentration of soluble compounds in pig slurry rose after hydrothermal pre-treatment below 90°C, increasing methane yield (Bonmatí et al., 2001).

**FIGURE 2:**
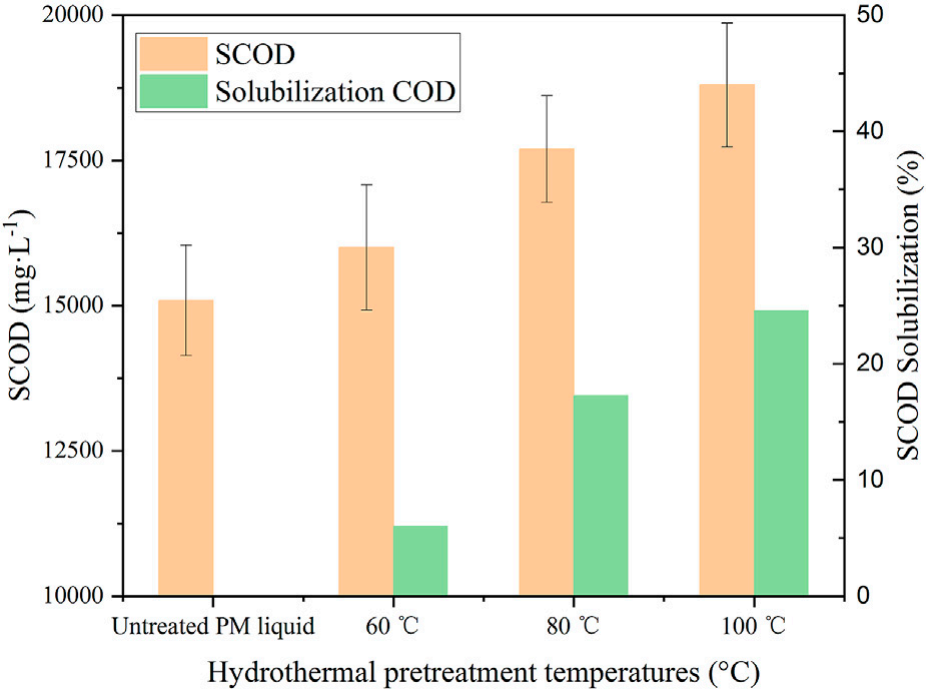
Enhancement of SCOD and COD solubilization by HPT.

The increment of solubility after pre-treatment can be expressed by solubilization COD which was calculated as Eq. 4 (Vlyssides and Karlis, 2004). As shown in Figure 2, all the SCOD of PM feed increased with increasing pre-treatment temperature under HPT. Pre-treatment at 100°C resulted in the maximum solubilization COD (24.57%), followed by pre-treatment at 80°C (17.25%), while pre-treatment at 60°C only slightly increased by 6.02%. Saragih found that heat pre-treatment of food waste at 70°C increased SCOD and solubilization by 10.2% and 24.7%, respectively (Saragih et al., 2019). Passos studied the HPT in microalgae, finding that pre-treatment at 75°C and 95°C significantly improved the soluble matter content and biogas yield of microalgae. In comparison, that at 55°C only resulted in slight increases in both parameters (Passos et al., 2013). Dhar also observed that pre-treatment at 70°C increased the SCOD/TCOD ratio by 18%–19% in the sample and that at 90°C increased the ratio by 35%–37% (Dhar et al., 2012). The results of the present study and previous literature indicate that heat pre-treatment at a temperature above 70°C can promote the dissolution of the solid matter. However, the temperature has a different influence on the SCOD of different materials, which may be ascribed to the biomass’s different characteristics (composition, TS and VS/TS content) (Ruffino et al., 2015).

Additionally, the growth of SCOD obtained by HPT in the present study was lower than that obtained from the pre-treatment under high temperature and pressure (Usman Khan and Kiaer Ahring, 2021), indicating that short-time HPT has little effect on the hydrolysis of recalcitrant organic compounds. Kamaraj found that hemicellulose and cellulose can only be effectively hydrolyzed at high temperatures such as 150–180°C, and cellulose is generally hydrolyzed slowly or sometimes even not hydrolyzed (Kaparaju et al., 2009).

### BMP test

**FIGURE 3:**
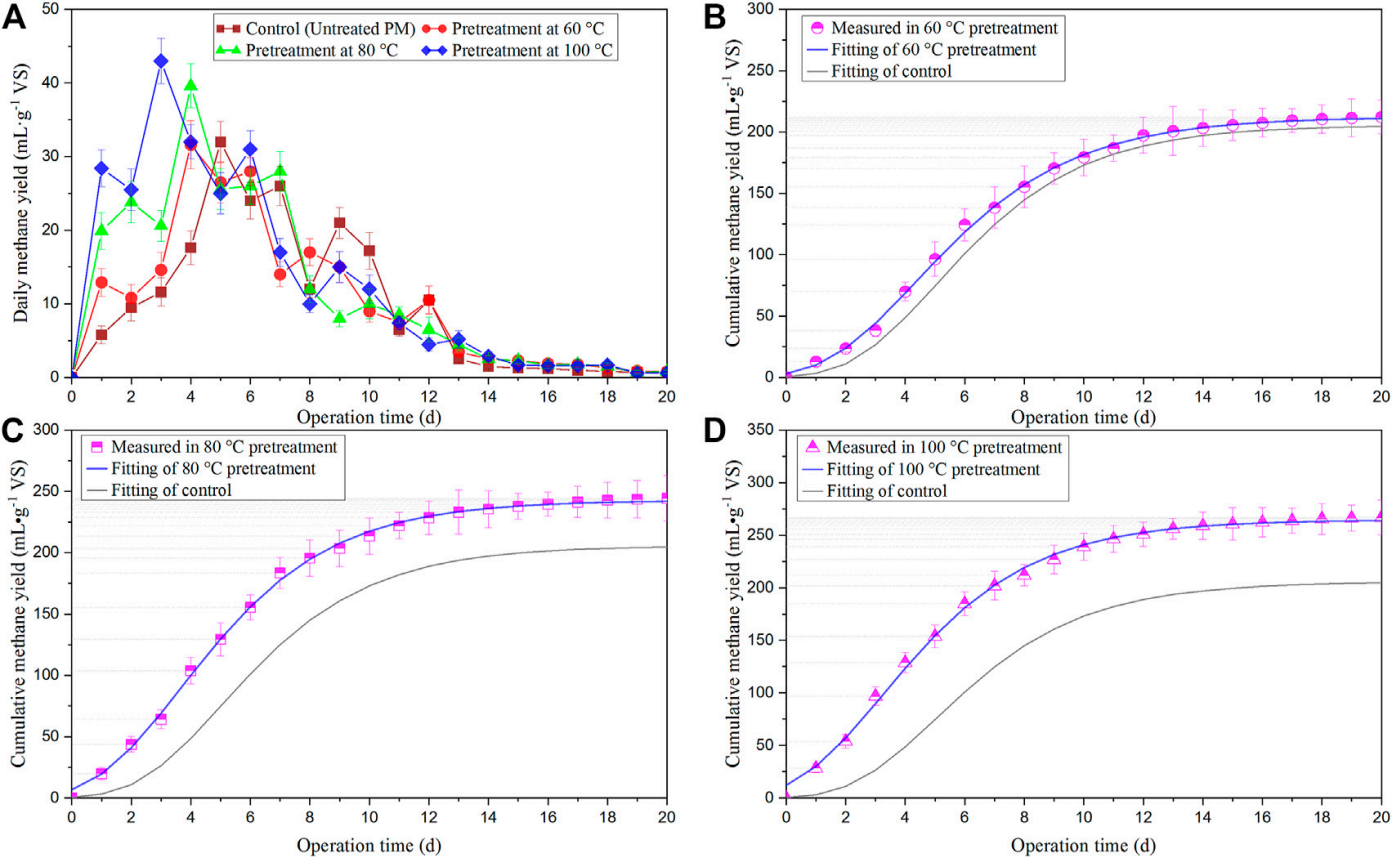
Daily methane yields **(A)** and the measured cumulative methane yields with their fitting curves **(B–D)** in the BMP test of PM feed.

Figure 3 presents the daily methane yield (a) and the deviation of the measured cumulative methane production (scatter) from its fitted curves (solid line in b, c, and d), and the fitting results are summarized in Table 2. All the fit curves’ determination coefficients (R2) were higher than 0.99, indicating that the model was well-fitted. As shown in Figure 3, all the BMP tests had 20 days, after which the daily biogas production dropped below 1% of the cumulative production (VDI 4630, 2006). As shown in Figure 3A, the daily biogas production of pre-treated and untreated PM had a biogas production duration between 1 and 12 days. HPT promoted the initiation of the digestion process, resulting in much higher daily biogas production in the first days of the test. This can also be reflected by the shorter delay time of gas production (*λ*) under 80°C and 100°C pre-treatments (Table 2). Although an initial lag phase in methane production was observed in all of the tests, the methane production started immediately on the first day of all the pre-treatment digestion (Li et al., 2016). Scarcelli indicated that when thermal pre-treatment was applied to the substrates, the methane yield increased, especially in the first few days, due to a higher share of soluble COD (Scarcelli et al., 2020).

**TABLE 2:** Fitting results of methane production kinetics in BMP test.

Pretreatment TEMP	*A*/mL·g^−1^ VS		*μ*_m_/mL·g^−1^·d^−1^		*λ*/d		*R* ^2^
	Estimated value	Std. Deviation	Estimated value	Std. Deviation	Estimated value	Std. Deviation	
Control	205.53	1.25	26.77	0.64	2.21	0.09	0.9992
60°C	212.34	1.12	25.82	0.52	1.34	0.08	0.9985
80°C	242.7	1.28	31.08	0.69	0.78	0.09	0.9991
100°C	264.64	1.75	34.02	0.98	0.36	0.12	0.9983

As shown in Table 2, the PM feed pre-treated at 60°C, 80°C, and 100°C had 3.31%, 18.09%, and 28.76% higher methane yields than the untreated PM feed, respectively. And the *μ*_*m*_ values obtained from 80°C to 100°C pre-treatments were higher than the control. This indicates that the pre-treatment at above 80°C can greatly improve anaerobic digestion and conversion efficiency (*p* < 0.05), which is consistent with some previous conclusions. For instance, Rafique found that pre-treatment at 100°C significantly increased the maximum methane production potential of dehydrated PM, while that at 50°C and 70°C had no such noticeable effect (Rafique et al., 2010). Passos reported that only pre-treatment at a higher temperature of 95°C could achieve a higher biogas production of microalgae (Passos et al., 2013). Gnaoui proved that food waste pre-treated at 100°C for 30 min showed a methane yield improvement of 23.68% compared to the control (Gnaoui et al., 2020). In addition, Appels discovered that pre-treatment at 70°C slightly decreased the efficiency of the subsequent anaerobic digestion of sludge, but pre-treatment at a higher temperature would significantly increase biogas production (Appels et al., 2010). However, some studies found that heat pre-treatment at a low temperature of 65°C or 70°C can also improve biogas yield (Sutaryo et al., 2014; Ruffino et al., 2015; Lin et al., 2021). Besides, other studies have also pointed out that pre-treatment below 100°C has a negligible effect on the final methane production, even if it promotes COD solubilization. Raju found that PM improved biogas production at pre-treatment temperatures of 125°C, while pre-treatment at 100°C did not improve. They also revealed that LTPT has little effect on the cellulose and hemicellulose fractions (Raju et al., 2013). Carrère proved that pre-treatment of 70–90°C can only increase the soluble substances and biogas production of the liquid part of PM while improving the overall biogas production need a higher temperature of >150°C (Carrère et al., 2009). The main reason for these discrepant findings may be that HPT is affected by various factors such as treatment time, substrate composition, and liquid TS.

### Semi-continuous AD test

The semi-continuous AD mode is generally used in practical engineering, and the parameter of volumetric biogas production rate (VBPR, L·L^−1^·d^−1^) is often employed to evaluate the output efficiency of a continuous AD tank (Liu et al., 2012; Li et al., 2015), mainly because the AD tank accounts for a large proportion of the engineering investment and high operation energy consumption for heat preservation and mixing (Mahmoodi-Eshkaftaki et al., 2017). Besides, the parameter of biogas production rate (BPR, mL∙g^−1^ VS_added_) is also crucial for calculating the cost because it can represent the utilization efficiency of raw materials. Hence, both parameters were considered better to evaluate the AD performance in a semi-continuous process.

**FIGURE 4:**
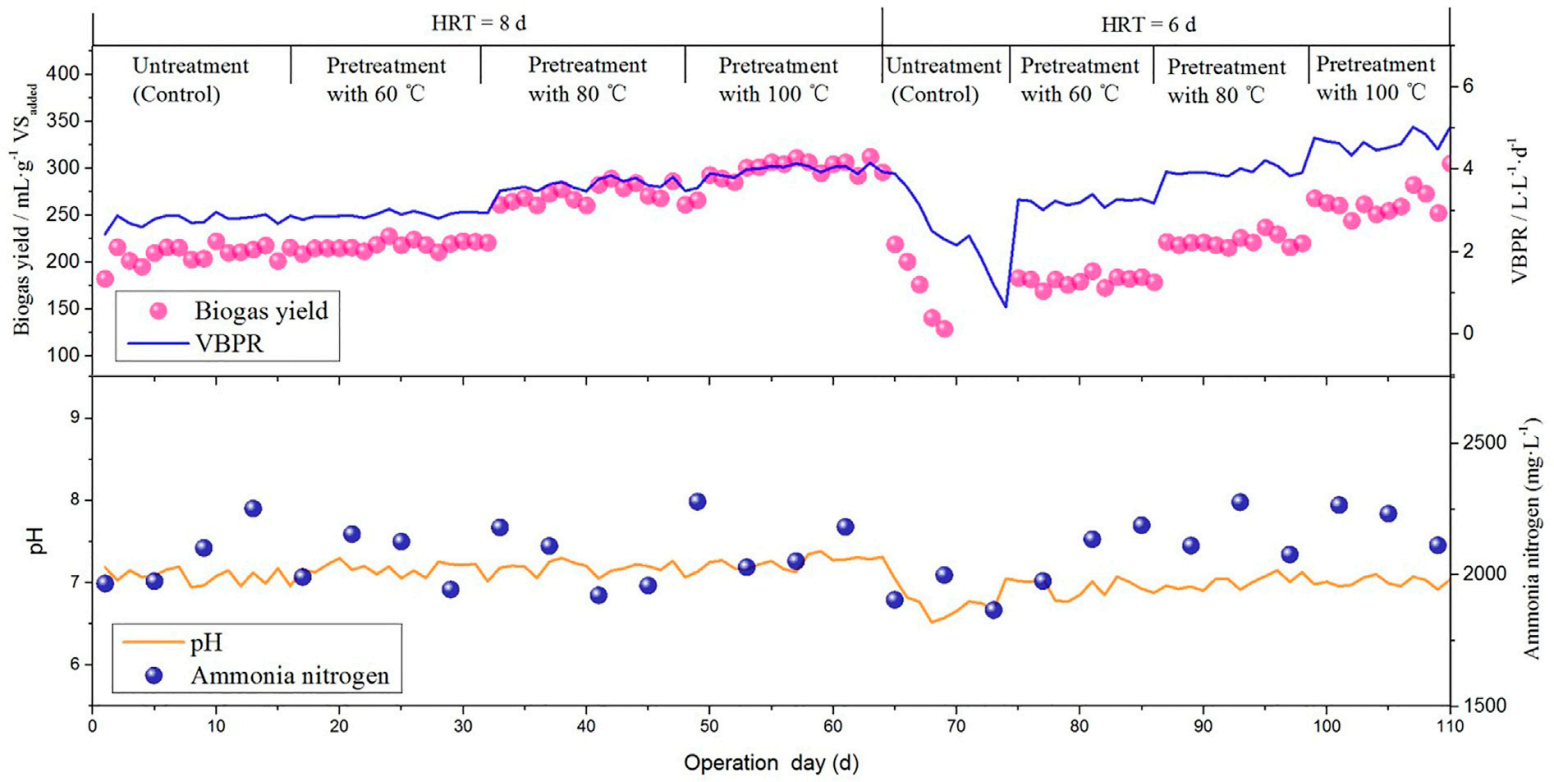
Changes of daily biogas production, pH and ammonia nitrogen in semi-continuous AD test.

As shown in Figure 4, the whole process can be divided into two phases (phase I and phase II) according to the different operations of HRT. In each phase, VBPR and BPR showed an upward trend with replacing untreated PM with heat pre-treated PM in digester feeding. However, the obtained specific values were quite different between the two phases. The maximum VBPR of 4.71 L L^−1^·d^−1^ (OLR = 17.8 g VS_added_·L^−1^·d^−1^) and the highest BPR of 297.9 ml g^−1^ VS. (OLR = 13.4 g VS_added_·L^−1^·d^−1^) were observed from the PM pre-treated by 100°C in phase II and phase I, respectively. Moreover, when operating under HRT for 8 days, all treatments resulted in a stable biogas production performance, and pre-treatment at 60°C, 80°C, and 100°C resulted in 4.3%, 30.6%, and 43.0% higher average VBPR than the control, respectively. When HRT was reduced to 6 days, the feeding of untreated PM resulted in a sharp decrease in biogas production. Sánchez observed that when the OLR increased up to 7 g VS.·L^−1^·d^−1^, a drastic reduction in the VS removal rate was found in the mesophilic semi-continuous anaerobic digestion of swine waste (Sánchez et al., 2021). However, in the present assay, the digester was paused for some time to buffer and then run using heat pre-treated PM feed. As a result, biogas production was recovered. This indicates that the digester will have a stronger capacity to bear higher OLR upon feeding heat pre-treated PM. Guo also revealed that co-digestion of heat pre-treated dewatered activated sludge and other municipal biowastes can significantly improve the ability of the digester to withstand high OLR and VFA accumulation (Guo et al., 2014).

With decreasing HRT from 8 days to 6 days, all the VBPR showed an increasing trend, while BPR was just the opposite. The maximum VBPR of 4.71 L L^−1^·d^−1^ (OLR = 17.8 g VS_added_·L^−1^·d^−1^) and the highest BPR of 297.9 ml g^−1^ VS (OLR = 13.4 g VS_added_·L^−1^·d^−1^) were observed from the PM feed pre-treated by 100°C, respectively. This result is consistent with some previous studies. Zhou proved that the volumetric methane production rate (0.25–5.69 L L^−1^·d^−1^) increased with increasing OLR (Zhou et al., 2022). Mazareli claimed that a higher OLR improved the volumetric methane production of swine wastewater (Mazareli et al., 2016). Increasing OLR can improve the biogas production of the semi-continuous AD tank but will also decrease its capacity to convert materials. I found that the VBPR of co-digested straw and manure increased with increasing OLR, but it was different for the BPR (Li et al., 2015). Tassakka optimized the OLR in the AD process of food waste and found that a higher OLR of 10 kg VS.·m^−3^·d^−1^ can achieve the highest biogas production, while a further increase in OLR will lead to a decrease in VS. conversion (Tassakka et al., 2019).

Table 3 shows that the BRR values range from 49.56% to 66.32%, indicating that a part of organic matters were not effectively utilized in semi-continuous AD process, and higher OLR and shorter HRT would correspond to lower BRR, which can be mainly ascribed to the characteristics of the semi-continuous AD mode. Under HRT of 8 days, all the BRR values of heat pre-treatments were higher than those of the control, suggesting that heat pre-treatment can promote the substrate conversion rate. Luste and Luostarinen found that pasteurization (70°C, 60 min) increased both the soluble substance and bioavailability of the mixture of slaughterhouse waste and sludge and enhanced the maximum methane yield by 24% compared with the control in a continuous AD process (Luste and Luostarinen, 2010). This can also be explained by the accumulation of VFAs, as the control had higher VFA accumulation than the heat pre-treatment groups.

**TABLE 3:** Performance of biogas production in semi-continuous AD process.

Parameters	Phase I: HRT of 8 days				Phase II: HRT of 6 days		
	Control	60°C	80°C	100°C	60°C	80°C	100°C
pH of effluent	7.08	7.20	7.30	7.43	6.84	7.05	7.14
AN of effluent/mg·L^−1^	2,248	2,319	2,513	2,700	1908	2,350	2,508
VFAs of effluent/g·L^−1^	2.35	1.79	1.12	0.98	1.69	1.41	1.18
BPR/mL·g^−1^ VS_added_	208.3	217.6	271.9	297.9	180.2	220.1	264.4
VBPR/L·L^−1^·d^−1^	2.77	2.89	3.62	3.96	3.21	3.92	4.71
CH_4_/%	54.5	55.4	57.2	56.3	56.4	55.2	56.9
BRR/%	57.26	58.82	66.32	65.63	49.56	51.87	58.85

Additionally, it has been found that the AN exceeding 3,000 mg L^−1^ will lead to a toxic effect on the methanogens (Calli et al., 2005), while all the AN values observed in the present study were lower than this threshold (Figure 4; Table 3). It is also worth noting that pH fluctuations are present throughout the semi-continuous AD process. However, it is still stable in a small area except for the stage when HRT decreased to 6 days to start feeding untreated pig manure liquid, which may be due to the relatively large volume of daily feed; bringing in more oxygen often causes reactor fluctuations.

### Energy balance analysis

Although thermal pre-treatment is an available approach used in the pilot- and full-scale implementation (Millati et al., 2020), it also needs to consume energy. Thus, considering pre-treatment’s energy balance is necessary to evaluate its efficiency and benefits (Marsolek et al., 2014). Under most tested conditions, the extra biogas production is insufficient to offset the energy required in pre-treatment (Cho et al., 2013; Ometto et al., 2014). The energy ratios (*E*_*i*_ */E*_*o*_) are summarized in Table 4, where values below 1 indicate a positive energy balance. It can be seen that all treatments can obtain a positive energy yield (*E*_*i*_ */E*_*o*_ < 1) running at HRT of 6 days. However, running at HRT of 8 days and without considering the heating energy recovery, no positive energy gain can be obtained for both 60°C and 100°C pre-treatment. This is not quite consistent with previous studies. Carrillo-Reyes reported the longest HRT of 30 days resulted in a positive energy balance, while a short HRT of 15 days showed a negative balance (Carrillo-Reyes et al., 2021). Sun also proved that mesophilic conditions operated in the longest HRT of 30 days obtained the highest *E*_*o*_ */E*_*i*_ (Sun et al., 2014). Moreover, the obtained *E*_*i*_ */E*_*o*_ values are lower than those reported in previous literature (Passos et al., 2013; Ometto et al., 2014). This is the advantage of HSAD mode, which has the potential to obtain high VBPR so as to achieve a higher energy output, while high water contents in organic substrates have been identified as a main factor for the excessive energy consumption during the pre-treatment (Tang et al., 2010).

**TABLE 4:** Energy expenditure and income of an assumed semi-continuous AD process.

Parameters	Phase I: HRT of 8 days				Phase II: HRT of 6 days		
	Control	60°C	80°C	100°C	60°C	80°C	100°C
^a^Daily PM feed volume/L	0.125	0.125	0.125	0.125	0.167	0.167	0.167
VBPR/L·L^−1^·d^−1^	2.77	2.89	3.62	3.96	3.21	3.92	4.71
*ΔP*/L	—	0.12	0.85	1.19	0.44	1.15	1.94
*E*_*o*_/kJ	—	2.23	15.23	21.46	7.88	20.66	34.85
*E*_*i*_/kJ	—	2.61	13.06	23.51	3.49	17.45	31.41
*E*_*i*_/*E*_*o*_	—	1.17	**0.86**	1.10	**0.44**	**0.84**	**0.90**
^b^ *E*_*i*_/kJ	—	—	6.53	11.76	—	6.98	12.57
^b^ *E*_*i*_/*E*_*o*_	—	—	**0.43**	**0.55**	—	**0.34**	**0.36**

^a^ Calculated as working volume of 1L divided by HRT.

^b^ Represents that a heat exchanger was used for energy recovery in HPT, and the heat energy recovery rates (*φ*) of pre-treated at 80°C and pre-treated at 100°C are calculated as 50% and 60% respectively.

Bolded values represent positive energy balance.

**FIGURE 5:**
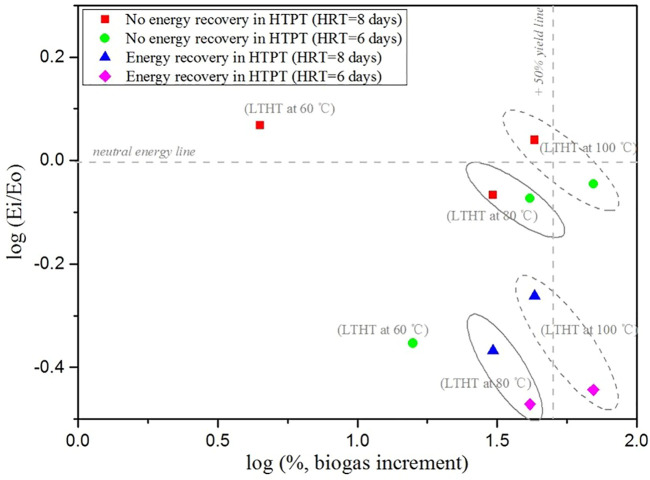
Energy balance of all the treatments and the related increment in biogas production.

Additionally, the observed *E*_*i*_ */E*_*o*_ values without energy recovery in HPT are higher than that in energy recovery heating mode. This is attributed to the high energy demand for heating PM feed. It confirms that it is necessary to recover part of the energy in HPT with a heat recovery device such as a heat exchanger. Figure 5 shows the data plotted on a log-log scale chart (*E*_*i*_/*E*_*o*_ ratio vs. biogas increment) for different treatments. The log *E*_*i*_/*E*_*o*_ values of pre-treatment with energy recovery in HPT are located at the bottom right part of the graph, representing a better energy balance than the control group. While the log *E*_*i*_/*E*_*o*_ values of pre-treatment without energy recovery in HPT are located near the neutral energy line, representing a weak energy balance. This indicates that adopting heat exchange to recover energy is necessary for the preheating process.

Cho and Ometto found that it is difficult to obtain a positive energy balance in microalgae AD process with heat pre-treatment, and attributed this phenomenon to the release of lower energy content compounds compared with those released after ultrasound and enzymatic hydrolysis (Cho et al., 2013; Ometto et al., 2014). Thus, whether to use heat pre-treatment in actual production, this also often depends on the biomass material characteristics. Besides, it also should consider the influence of heating temperature, exposure time (González-Fernández et al., 2012), material particle size (Menardo et al., 2012), chemical catalyst (Seyed Abbas et al., 2018), and the AD operation conditions on the results of energy balance.

## Conclusion

The present study investigates HPT’s effects on AD of high-solid PM feed for biogas production. HPT promotes the dissolution of particulate matter, resulting in the maximum solubilization COD of 24.57% at 100°C pre-treatment, followed by pre-treatment at 80°C (17.25%). In comparison, pre-treatment at 60°C only slightly increased by 6.02%. Similarly, the maximum methanation potential of 264.64 ml g^−1^ VS was obtained with 100°C pre-treatment, that in 80°C pre-treatment is 242.7 ml g^−1^ VS, they showed an increase of 28.76% and 18.09% compared to control, respectively. And the methanation potential of 60°C pre-treatment increased by only 3.31%. In the continuous test, HPT can reduce the impact of large-volume feed on the anaerobic system, which helps the reactor operate under shorter HRT or higher OLR to obtain a greater VBPR. The VBPR increased by 30.69% and 70.04% in the pre-treatment groups at 80°C and 100°C, respectively. This leads to *E*_*i*_ */E*_*o*_ values as low as 0.43–0.55, which shows that heat pre-treatment can get a positive energy balance in the HSAD of PM with high OLR.

## Data Availability

The original contributions presented in the study are included in the article/Supplementary Material, further inquiries can be directed to the corresponding author.
